# Evaluation of the effect of two conventional and one herbal mouthwash on the modulus of elasticity of three orthodontic wires: an in vitro study.

**DOI:** 10.12688/f1000research.156706.1

**Published:** 2025-03-27

**Authors:** Neharika Kapur, Ritesh Singla, Nishu Singla, Madhumitha Natarajan

**Affiliations:** 1 Department of Orthodontics & Dentofacial Orthopaedics, Manipal College of Dental Sciences, Manipal, Manipal Academy of Higher Education, Manipal, Karnataka, 576104, India; 2 Department of Public Health Dentistry, Manipal College of Dental Sciences, Manipal, Manipal Academy of Higher Education, Manipal, Karnataka, 576104, India

**Keywords:** Modulus of Elasticity (MoE), stiffness, orthodontics archwires, Stainless steel, nickel-titanium, Beta titanium, Mouthwash, chlorhexidine, sodium fluoride, herbal, HiORA.

## Abstract

**Background:**

During fixed appliance therapy, maintaining good oral hygiene is crucial; however, certain mouthwashes can negatively impact orthodontic treatment by altering the mechanical properties of archwires. The study investigated the impact of conventional mouthwashes on orthodontic archwires' Modulus of Elasticity (MoE).

**Methods:**

It was an in vitro study that tested MoE of stainless steel (SS), nickel-titanium (NiTi), and β-titanium (β-Ti) archwires after immersing them in chlorhexidine (CHX), sodium fluoride (NaF), and HiORA mouthwashes along with distilled water (control) for one minute daily over three months. The wires were then subjected to a three-point bending test to measure their flexural modulus of elasticity (MoE) while applying loading and unloading forces at 0.5, 1, 1.5, 2, and 2.5 mm deflections. Statistical analysis included two-way ANOVA and Tukey’s Post Hoc test at significant α≤0.05.

**Results:**

NaF reduced MoE for SS (loading 172.99 ± 7.71 GPa, unloading 148.88 ± 14.65 GPa), NiTi (Loading: 48.42 ± 6.13 GPa, unloading 30.60 ± 4.29 GPa), β-Ti wires (unloading 79.51 ± 1.62 GPa) than Control (loading 185.35 ± 8.06 GPa, unloading 199.78 ± 9.49 GPa) with p<0.05. CHX mouthwash reduced SS (loading -158.58 ± 4.47 GPa, unloading-138.54 ± 6.12 GPa), NiTi (unloading 37.05 ± 2.58 GPa), β-Ti (unloading 81.58 ± 7.94 GPa) with p<0.05. HiORA showed no significant differences in MoE for SS (loading 190.28 ± 7.54 GPa, unloading 185.58 ± 5.35 GPa), NiTi (loading 58.85 ± 2.39, unloading 37.05 ± 2.58), or β-Ti archwire (loading 89.92 ± 2.20, unloading 104.22 ± 5.00) compared to the controls with p<0.05.

**Conclusion:**

HiORA herbal mouthwash is safe for NiTi, SS, and β-Ti archwires at all stages. Chlorhexidine (CHX) mouthwash should be prescribed briefly to prevent MoE degradation in NiTi and β-Ti archwires. NaF-based rinses should be cautiously used and monitored in SS, NiTi, and β-Ti archwires patients.

## Introduction

Fixed orthodontic appliances create areas where plaque can accumulate more quickly, especially around brackets and wires. Plaque build-up around orthodontic appliances usually leads to gingival inflammation and bleeding. If the plaque is not removed correctly, it can also lead to the demineralization of tooth enamel and the development of white spot lesions, which can be permanent and aesthetically displeasing. Orthodontic patients must maintain oral hygiene to promote gingival health and prevent white spot lesions or tooth decay, leading to healthier and more successful orthodontic treatment outcomes. Hence, patients are often advised to use mouthwash as it can help reduce plaque accumulation in areas that are difficult to clean with a toothbrush or floss. It can also aid in remineralizing white spots and preventing tooth decay. ^1^

Mouthwashes often contain various chemicals, including alcohol, chlorhexidine, fluoride, and other antimicrobial agents. Prolonged exposure to the chemicals in the mouth can corrode or degrade orthodontic wires made of metal alloys such as stainless steel, nickel-titanium, or beta-titanium and alter their mechanical properties. ^2– 5^ This can increase surface roughness, promoting bacterial adhesion and plaque accumulation. ^6^ Changes in the surface properties of orthodontic wires can also affect friction between the wires and brackets, impacting tooth movement and treatment efficiency. ^7^ The Modulus of Elasticity (MOE) measures a material's stiffness or rigidity. Certain mouthwashes can affect the MOE of orthodontic wires, making them more susceptible to deformation or breakage when subjected to applied forces during orthodontic treatment. ^2^ This can complicate orthodontic treatment by causing discomfort or delaying the treatment. Many studies have shown that using mouthwashes that contain active ingredients like NaF/CHX while undergoing orthodontic treatment can degrade the mechanical properties of the archwires. ^2– 14^

An archwire's modulus of elasticity is directly proportional to the magnitude of force delivered by an appliance. ^15^ Alloys with a high modulus of elasticity, such as stainless steel, are more rigid. Those with a low modulus of elasticity, such as nickel-titanium (NiTi), are more flexible. ^16^ Nickel-titanium alloys used as archwires have mechanical properties that allow them to exert gentle, continuous forces with a broader range than those of stainless steel. During the alignment phase, low-stiffness archwires like NiTi are preferred as they permit the application of less and a more consistent force over some time, and they maintain accuracy while applying a given force. On the other hand, a wire with a high stiffness and high spring back can resist distortion due to extra and intra-oral fractional forces, as seen in SS wires. This enables its use for significant tooth movements, including closure of spaces. However, when the clinical situation requires a wire with stiffness lower than SS while resisting deformation, i.e., more than NiTi, the β-Ti archwire can be used in the orthodontic treatment's finishing and detailing stage. ^5, 16^ Thus, precise knowledge of the MoE of the archwire is helpful during treatment.

Orthodontic patients should be careful when choosing mouthwashes for their treatment. It is crucial to consult with an orthodontist to receive recommendations on how to avoid any negative impacts. Orthodontists may suggest using alcohol-free, non-abrasive mouthwashes explicitly formulated for orthodontic patients to minimize adverse effects on treatment outcomes. ^17^ Herbal mouthwashes can be a good option for orthodontic patients due to their natural ingredients, such as herbs, essential oils, and plant-based extracts, which make them safer than mouthwashes that contain chemicals. HiOra mouthwash (a product by Himalaya Wellness) is an herbal mouthwash intended to improve oral hygiene. ^18^ It comprises herbal extracts of (Belleric myrobalan (Bibhitaki), Salvadora persica (Meswak), Tulsi, Oaktree Triphala (a mixture of Amalaki, Haritaki, Vibhitaki), Charmomile, Ocimum, Terminalia chebula, Celery, Licorice, Spearmint, Neem, Yavani, Satva, Nagavalli, Gandhapura taila, pilu, Peppermint satva, Bakula, Katha (astringent) ela, etc. ^19^ However, it's essential to review the ingredients to ensure that no components in this herbal mouthwash may interfere with orthodontic treatment. Currently, no research has been conducted to determine the impact of this herbal mouthwash on orthodontic wires.

This study was conducted to identify a safe preventive agent for regular use in patients undergoing orthodontic treatment. This study aimed to examine the influence of conventional mouthwashes such as Chlorhexidine, Sodium Fluoride, and HiORA herbal mouthwash while Distilled water as a control on the stiffness of routinely used preformed orthodontic archwires made of stainless steel (SS), β-titanium (β-Ti), and nickel-titanium (NiTi). Modulus of Elasticity (MoE) is an essential metric for evaluating the stiffness or rigidity and load-deflection properties of orthodontic archwires, which may impact the clinical performance of the wires. The study findings are valuable for orthodontic treatment and research.

## Methods

This was an in vitro study conducted to examine the influence of conventional mouthwashes on the stiffness of routinely used preformed orthodontic archwires. The study was approved by the Kasturba Hospital Ethics Committee, Kasturba Hospital, Manipal, Karnataka (IEC 869/2019) on 13 ^th^ November 2019. The study tested three pre-formed orthodontic archwires of SS, β-Ti, and NiTi. The sample size was calculated based on the prior research, considering a 95% significance level, 95% confidence level, and 80% power. ^2^ There were six specimens for each of the three wire types across four test groups, resulting in 96 specimens for testing. Twenty-four specimens of each kind of wire (0.016” Niti, 0.019*0.025” SS, and 0.019*0.025” β-Ti- AO Sheboygan USA), 30 mm in length were cut from the straight part of the prefabricated maxillary orthodontic arch-wires.

**Figure 1.**
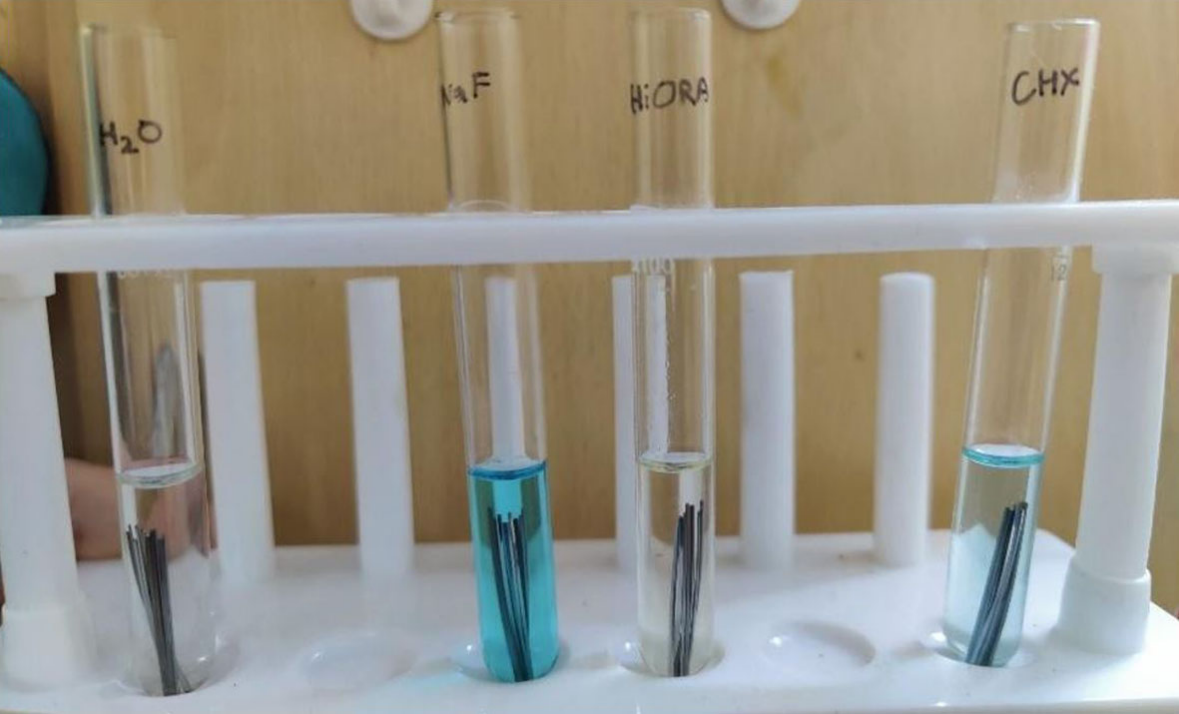
Borosil Glass Test tubes with wire specimens immersed.

Six specimens of each of the three wire types were immersed in glass test tubes containingfour different test solutions of 7ml each ( Figure 1).

Test Group I: 0.2% Chlorhexidine gluconate (CHX) mouthwash, ICPA Health products, India

Test Group II: 0.05% Sodium Fluoride (NaF) mouthwash, Colgate Oral Pharmaceuticals Inc., USA

Test Group III: HiOra herbal mouthwash, The Himalaya Drug Company, India

Test group IV: Distilled water (the control).

As per Walker et al., the specimens were immersed in the mouthwashes in glass test tubes for 1-minute daily application (30 seconds in the daytime followed by 30 seconds at night) for three months. ^4, 5^ After the immersion, each wire sample was removed from its respective solution, washed with distilled water, dried, and set aside in clean individual plastic Ziploc covers with the personal name tag and stored at room temperature.

**Figure 2.**
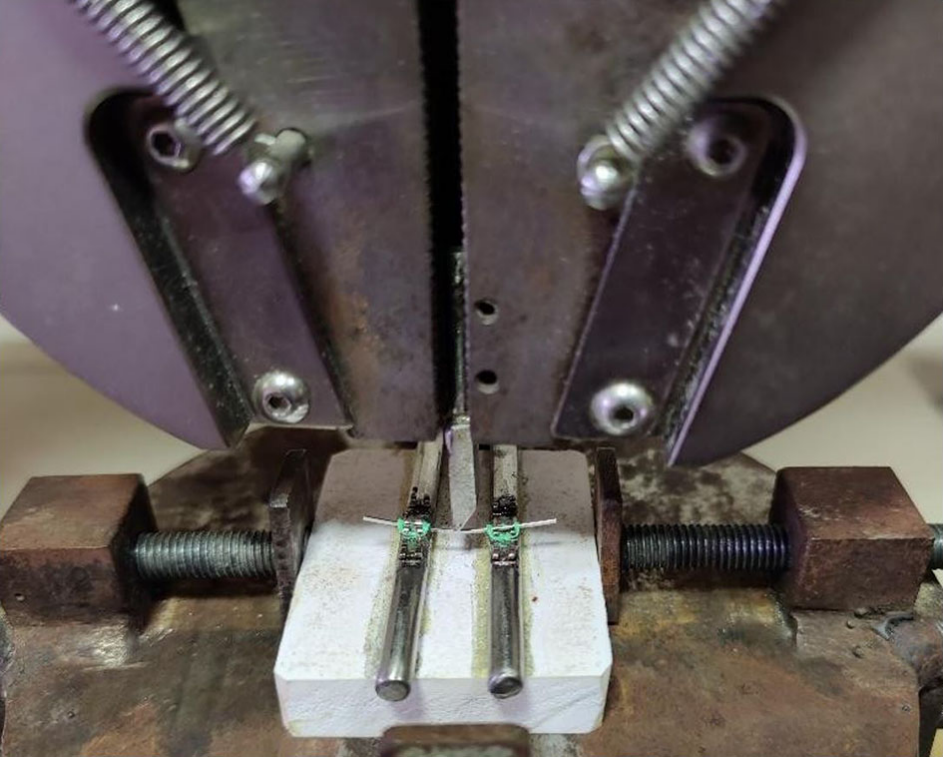
Customized Jig.

After three months of repeated immersion, a customized jig was made for the wires based on a study conducted by Aghili et al. ^2^ It consisted of 2 cylindrical metal rods bonded on top with two MBT stainless-steel brackets of 0.022 x 0.028” dimension with an inter-bracket distance of 15.5 mm. The wires were placed in the brackets and fixed with ligatures (3M Alastik ^TM^ modules) to keep them stable. It was mounted on top of rectangular acrylic blocks ( Figure 2). The 3-point fixture had a 15.5 mm support span, with the wire deflected in the middle, per Wilkinson's model. ^20^ In a male permanent dentition, It resembles the space between the maxillary central incisor bracket and the maxillary canine bracket on the same side. ^2^

**Figure 3.**
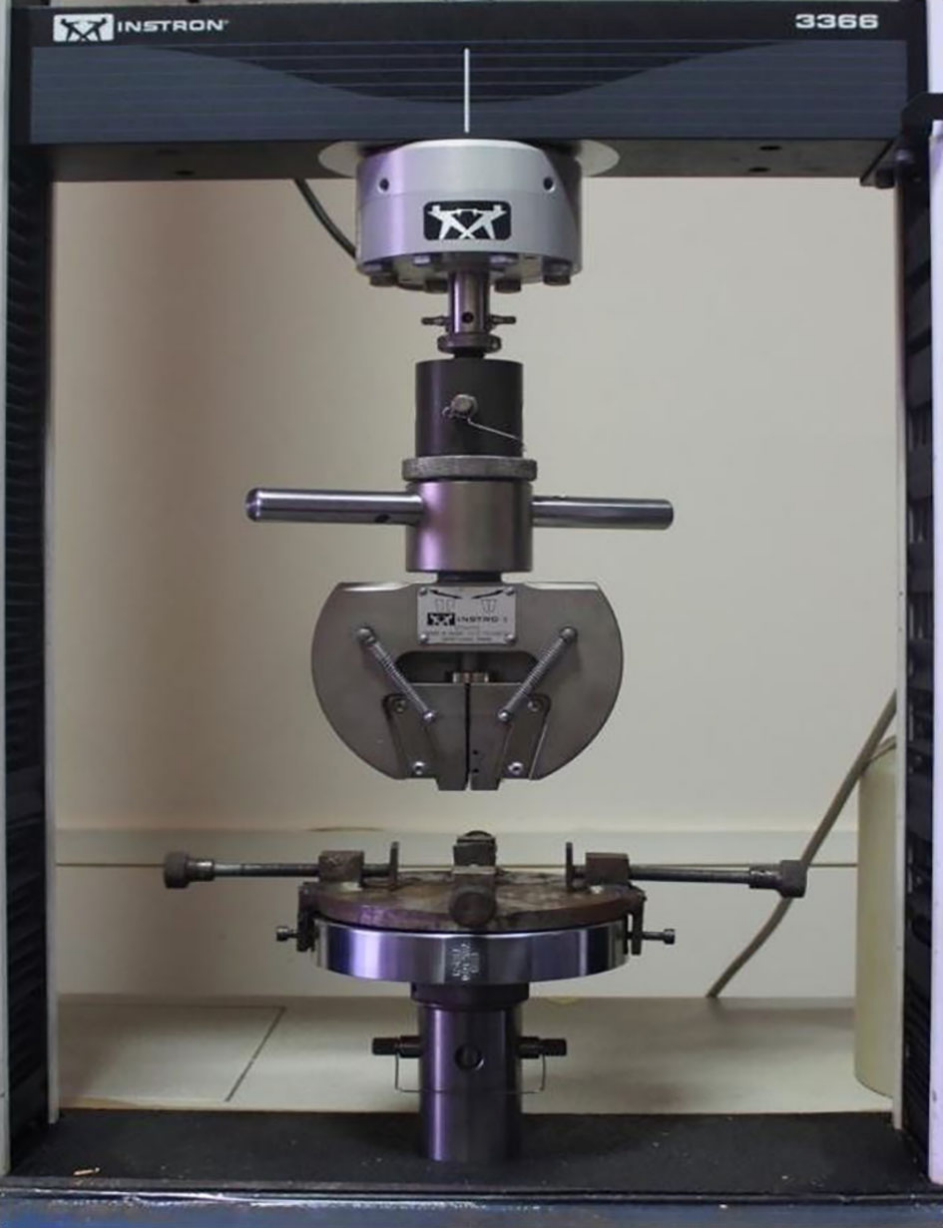
Universal Testing Machine (INSTRON 336).

All wire samples were subjected to a three-point bending test on the universal testing machine (Instron 336). Mechanical testing was based on American National Standard/American Dental Association Specification No. 32 for Orthodontic Wires ( Figure 3). ^21^ Blue Hill software recorded the loading values for each sample at 0.5, 1, 1.5, 2, and 2.5-mm deflections. Keeping the crosshead speed of 0.5 mm/min, the individual specimen was loaded to a maximum deflection of 2.5 mm and subsequently unloaded to zero deflection, taking 5 minutes for loading and 5 minutes for emptying. For loading and unloading, force in Newtons and deflection in millimeters were recorded for each specimen with a computer software program Bluehill. Using the specimen proportions and load-deflection curve, the slope of each archwire was determined at the first linearmost portion of the load-deflection curve (N/m).

**Figure 4.**
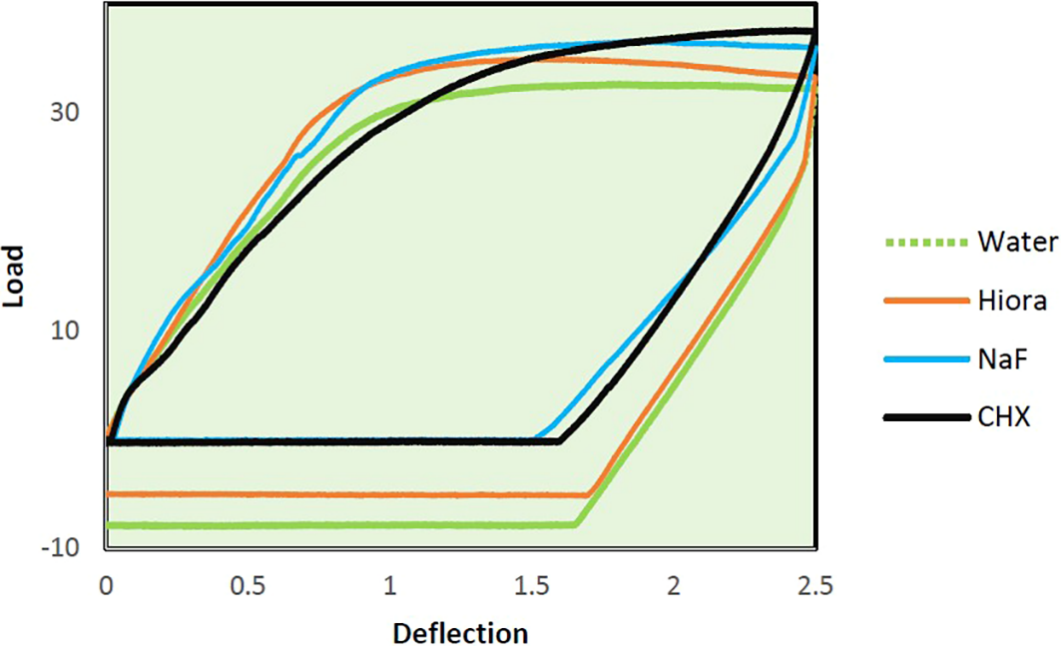
Load deflection curve of SS archwire samples.

**Figure 5.**
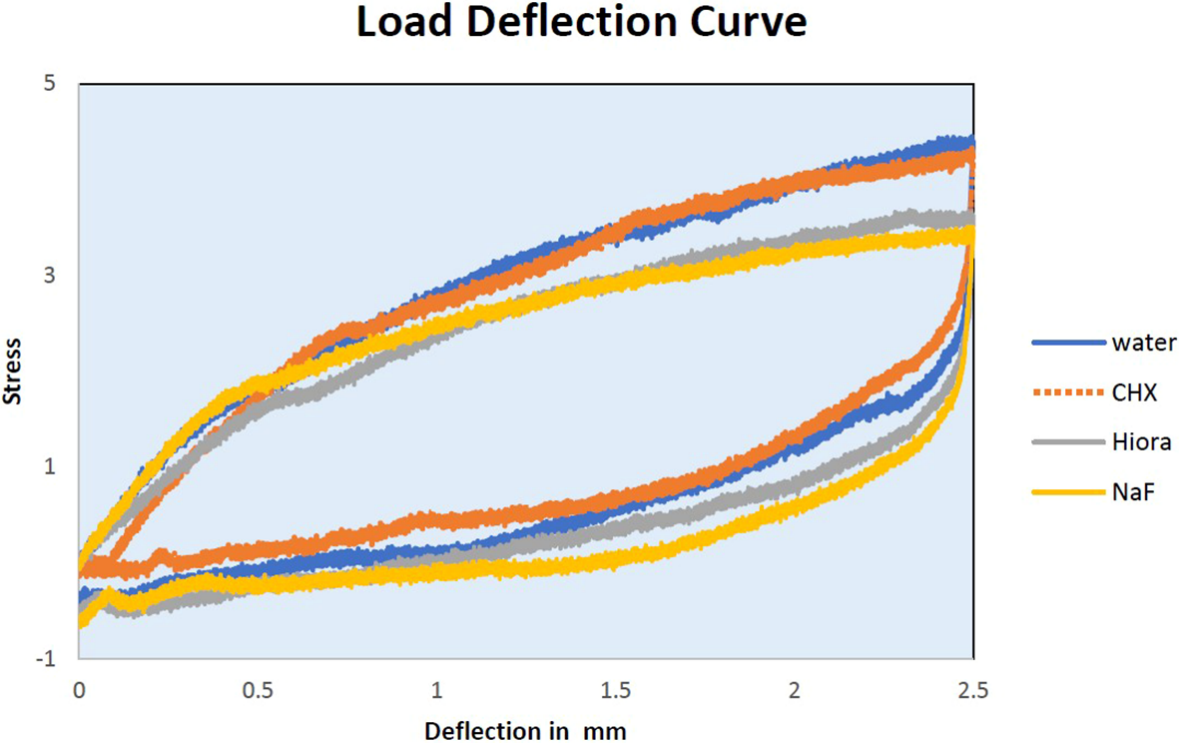
Load deflection curve of NiTi archwire samples.

**Figure 6.**
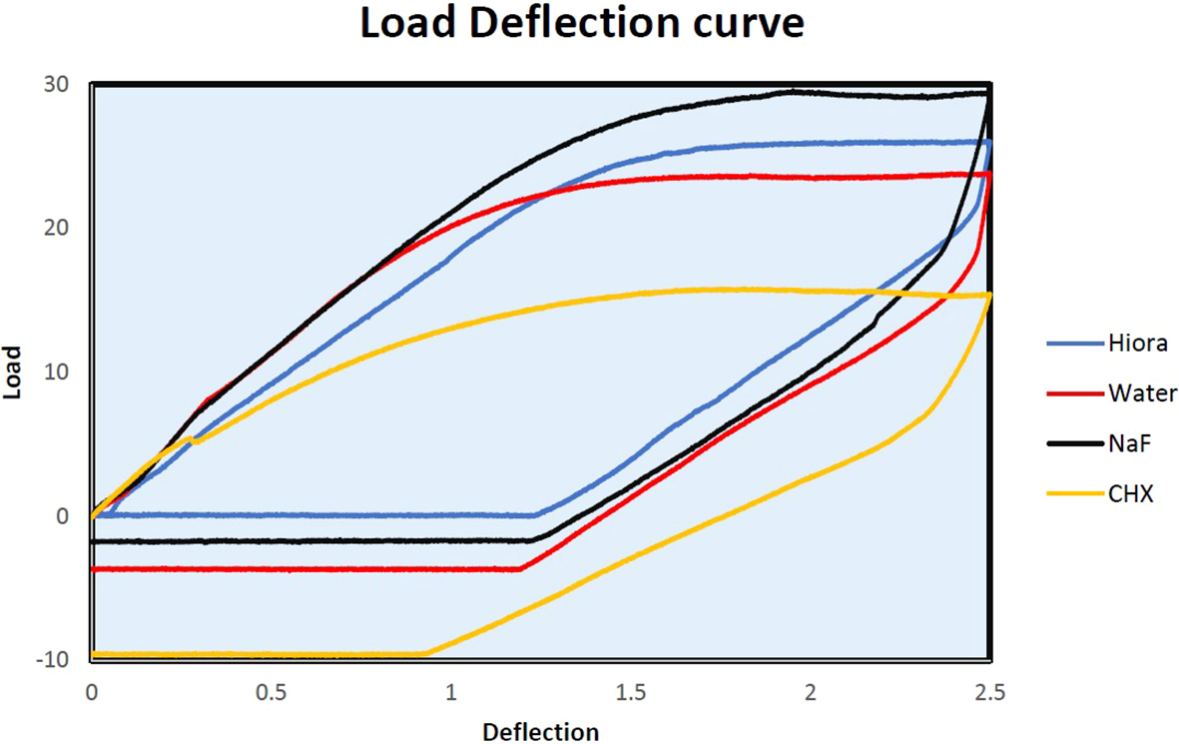
Load deflection curve of β-Ti archwire samples.

The slopes of Force-Deflection curves were utilized to measure the parameter of MoE for the examined wire samples (( Figures 4, 5, 6) The MoE for each wire sample was calculated using the following formulae. The formula for the rectangular archwires (namely SS and β-Ti) used was (L ^3^m)/4bd ^3,^ and the formula for the round NiTi archwires was L ^3^m/(6r ^3^d) where:

m= Load Deflection Curve of a given sample.

L = The distance between the 2 rods used to fix the sample archwire (mm).

b= width of archwire sample (mm).

d= depth of archwire sample (mm).

r = radius of round cross-section archwire (mm).

### Statistical analysis

Statistical software SPSS version 29 was used for the statistical analysis. The mean and standard deviation of loading forces (N) in all groups of the archwire in different displacement intervals at 0.5, 1, 1.5, 2, and 2.5 mm complied with descriptive statistics. The data's normality was evaluated using the Kolmogorov–Smirnov test, indicating a normal distribution (P>0.05), allowing for parametric tests. The loading forces and flexural MoE data were analyzed by one-way ANOVA followed by Tuckey’s Post Hoc test. The significance level was kept at α ≤ 0.05.

## Results

Tables 1 and 2 contain the values of the flexural MoE of each orthodontic wire, which varies depending on the composition of the wire and the mouthwash used. Table 3 compares the Mean and standard deviation of loading forces (N) in orthodontic archwires in different displacement intervals (mm) between the test and control groups.

**Table 1.** Comparison of Mean and standard deviation of MoE of Loading samples (in GPa) between the study groups.

Archwire	Mouthwash (Test)		Distilled Water (Control)	Mean difference	P value
**SS**	**CHX**	158.58 ± 4.47	185.35 ± 8.06	- 26.77	**<0.001**
	**NaF**	172.99 ± 7.71	185.35 ± 8.06	- 12.36	**0.041**
	**HiORA**	190.28 ± 7.54	185.35 ± 8.06	4.94	1
**NiTi**	**CHX**	71.02 ± 3.45	63.58 ± 4.52	7.44	**0.05**
	**NaF**	48.42 ± 6.13	63.58 ± 4.52	- 15.16	**<0.001**
	**HiORA**	58.85 ± 2.39	63.58 ± 4.52	- 4.73	0.444
** β-Ti**	**CHX**	85.17 ± 7.08	90.77 ± 5.02	- 5.61	0.996
	**NaF**	85.98 ± 10.12	90.77 ± 5.02	- 4.80	1
	**HiORA**	89.92 ± 2.20	90.77 ± 5.02	-0.85	1

P value was derived by one-way ANOVA followed by Tuckey’s Post Hoc test. P≤ 0.05 is considered a significant difference.

**Table 2.** Comparison of Mean and standard deviation of MoE of Unloading samples (in GPa) between the study groups.

Archwire	Mouthwash (Test)		Distilled Water (Control)	Mean difference	P value
**SS**	**CHX**	138.54 ± 6.12	199.78 ± 9.49	- 61.24	**<0.001**
	**NaF**	148.88 ± 14.65	199.78 ± 9.49	- 50.90	**<0.001**
	**HiORA**	185.58 ± 5.35	199.78 ± 9.49	-14.20	0.113
**NiTi**	**CHX**	37.05 ± 2.58	63.33 ± 1.56	- 26.28	**<0.001**
	**NaF**	30.60 ± 4.29	63.33 ± 1.56	-32.72	**<0.001**
	**HiORA**	65.33 ± 1.85	63.33 ± 1.56	2.01	1
** β-Ti**	**CHX**	81.58 ± 7.94	95.52 ± 5.78	-13.94	**0.002**
	**NaF**	79.51 ± 1.62	95.52 ± 5.78	-16.00	**<0.001**
	**HiORA**	104.22 ± 5.00	95.52 ± 5.78	8.70	0.81

P value was derived by one-way ANOVA followed by Tuckey’s Post Hoc test. P≤ 0.05 is considered a significant difference.

**Table 3.** Compares Mean and standard deviation of loading forces (N) in SS, NiTi, β-Ti archwire in different displacement intervals (mm) between the test and control groups.

Archwire	Mouthwash (Test)			Distilled Water (Control)	Mean difference	P value
**SS**	**CHX**	**0.5mm**	20.07±1.78	24.16±2.99	- 4.09	**0.02**
		**1mm**	25.60±2.49	28.97±0.95	- 3.37	**0.01**
		**1.5mm**	30.20±2.72	33.64±3.95	- 3.44	0.1
		**2mm**	31.20±2.71	34.07±4.56	- 2.87	0.2
		**2.5mm**	30.52±2.68	35.49±5.36	- 4.97	0.07
	**NaF**	**0.5mm**	18.42±2.30	24.16±2.99	- 5.74	**0.004**
		**1mm**	27.92±2.98	28.97±0.95	- 1.05	0.43
		**1.5mm**	34.03±2.49	33.64±3.95	0.39	0.85
		**2mm**	34.71±2.60	34.07±4.56	0.64	0.77
		**2.5mm**	34.97±2.73	35.49±5.36	- 0.52	0.84
	**HiORA**	**0.5mm**	23.13±3.55	24.16±2.99	- 1.03	0.60
		**1mm**	29.44±1.92	28.97±0.95	0.47	0.60
		**1.5mm**	32.45±2.00	33.64±3.95	- 1.19	0.53
		**2mm**	33.12±2.44	34.07±4.56	- 0.95	0.66
		**2.5mm**	35.95±2.70	35.49±5.36	0.46	0.85
**NiTi**	**CHX**	**0.5mm**	1.35±0.50	1.30 ±2.0	0.05	0.95
		**1mm**	2.33±0.45	2.03 ±1.6	0.3	0.67
		**1.5mm**	3.02±0.52	2.60 ±1.54	0.42	0.54
		**2mm**	3.52±0.60	2.94 ±2.04	0.58	0.52
		**2.5mm**	3.86±0.59	3.21 ±1.9	0.65	0.44
	**NaF**	**0.5mm**	1.15±0.14	1.30 ±2.0	- 0.15	0.86
		**1mm**	1.93±0.41	2.03 ±1.6	- 0.1	0.86
		**1.5mm**	2.39±0.51	2.60 ±1.54	- 0.21	0.76
		**2mm**	2.73±0.54	2.94 ±2.04	- 0.21	0.81
		**2.5mm**	2.90±0.58	3.21 ±1.9	- 0.31	0.71
	**HiORA**	**0.5mm**	1.28±0.05	1.30 ±2.0	- 0.02	0.98
		**1mm**	2.13±0.04	2.03 ±1.6	0.1	0.88
		**1.5mm**	2.76±0.01	2.60 ±1.54	0.16	0.80
		**2mm**	3.12±0.01	2.94 ±2.04	0.18	0.83
		**2.5mm**	3.30±0.13	3.21 ±1.9	0.09	0.91
** β-Ti**	**CHX**	**0.5mm**	7.37±0.83	10.79 ±1.50	- 3.42	**0.0006**
		**1mm**	11.22±1.34	17.61 ±2.0	- 6.39	**0.0001**
		**1.5mm**	12.72±1.63	19.15 ±2.1	- 6.43	**0.0001**
		**2mm**	13.06±1.49	20.19 ±1.7	- 7.13	< **0.0001**
		**2.5mm**	13.19±1.37	26.95±1.54	-13.76	**<0.0001**
	**NaF**	**0.5mm**	9.91±0.87	10.79 ±1.50	- 0.88	0.24
		**1mm**	18.18±0.46	17.61 ±2.0	0.57	0.51
		**1.5mm**	21.87±1.77	19.15 ±2.1	2.72	**0.04**
		**2mm**	22.49±2.18	20.19 ±1.7	2.3	0.07
		**2.5mm**	22.50±2.27	26.95±1.54	- 4.45	**0.003**
	**HiORA**	**0.5mm**	10.54±1.51	10.79 ±1.50	- 0.25	0.78
		**1mm**	19.21±1.68	17.61 ±2.0	1.6	0.16
		**1.5mm**	24.05±2.11	19.15 ±2.1	4.9	**0.003**
		**2mm**	25.09±2.54	20.19 ±1.7	4.9	**0.003**
		**2.5mm**	25.30±2.49	26.95±1.54	-1.65	0.20

P value was derived by one-way ANOVA followed by Tuckey’s Post Hoc test. P≤ 0.05 is considered a significant difference.

### Stainless steel orthodontic archwire

The mean MoE of SS wires was found to decrease significantly after being treated with CHX (Loading-158.58 ± 4.47 GPa, Unloading-138.54 ± 6.12) and NaF (Loading-172.99 ± 7.71 GPa, Unloading-148.88 ± 14.65), compared to distilled water (control, loading-185.35 ± 8.06 GPa, unloading-199.78 ± 9.49), indicating that these mouthwashes have altered the properties of the SS wire by reducing its stiffness while loading and unloading the forces. Although HiORA mouthwash (Loading-190.28 ± 7.53 GPa, Unloading- 185.58 ± 5.35) had a mild effect on the MoE of SS wires, its use did not cause a significant change in the mechanical properties of SS orthodontic wires.

Load deflection: When stainless steel (SS) is exposed to CHX and NaF, the force required to cause a deflection between 0.5 mm to 1 mm decreases compared to control SS. This implies that the mechanical properties of the archwire become weaker when treated with CHX and NaF mouthwash. However, for deflections between 1.5 mm and 2.5 mm, there is only a slight change in the loading forces compared to control SS. HiORA, on the other hand, shows no significant change in forces across all deflections.

### Nickel titanium orthodontic archwire

According to the study, when NiTi wires were immersed in CHX, their loading MoE (71.02 ± 3.45 GPa) was slightly higher than when immersed in distilled water (loading 63.05 ± 5.8 GPa). However, its MoE was significantly reduced while unloading the forces (37.88±1.76 GPa) compared to the control-distilled water (unloading 63.33 ± 1.56 GPa). On the other hand, when exposed to NaF, the NiTi archwire experienced a significant reduction in both loading MoE (48.42 ± 6.13 GPa) and unloading MoE (30.60 ± 4.29 GPa) as compared to distilled water (control). Hence, NaF significantly altered the properties of NiTi archwires by decreasing their stiffness. Interestingly, HiORA did not considerably affect the NiTi archwire's stiffness (loading MoE=58.85 ± 2.39 GPa and unloading MoE=65.33 ± 1.85).

Load deflection: As with the NiTi archwire, we can conclude that with the application of CHX, the loading forces needed for the same deflection as the control archwire have an insignificant increase. With NaF mouthwash, the loading forces required have deteriorated slightly. There is a negligible alteration in the HiORA group of samples as well.

### Beta titanium orthodontic archwire

The CHX and NaF mouthwash resulted in a significant reduction only in the unloading MoE of β-Ti wires (unloading CHX- 81.58 ± 7.94 GPa, unloading NaF- 79.51 ± 1.62 GPa) from the control wire sample (Loading MoE 90.77 ± 5.02 GPa and Unloading MoE 95.52 ± 5.78 GPa). On the other hand, HiORA did not considerably affect the MoE of the β-Ti archwire (Loading-89.92 ± 2.20 GPa, unloading- 104.22 ± 5.00 GPa).

Load deflection: Beta Titanium archwire with NaF showed a minor increase of loading forces between 1mm to 2.0 mm w.r.t control, whereas, with the CHX group, it shows a marked decrease of loading forces required for the same deflection from 0.5mm to 2.5mm. HiORA again was deemed to have a very gentle effect similar to control, whereas the increase of loading forces between 1.5mm to 2.0 mm to control. This implies that the mechanical properties of the archwire become weaker when treated with CHX mouthwash.

## Discussion

### Stainless steel orthodontic archwire

The current study's findings also confirmed that using mouthwashes containing CHX and NaF led to changes in the mechanical properties of SS orthodontic wire. The CHX has the most adverse effect on the MoE values, and the NaF mouthwash also had a significant impact on the MoE values of SS archwire but less in comparison to CHX. Furthermore, the loading force required to cause a deflection decreases when treated with CHX and NaF mouthwash, indicating weakened mechanical properties of the archwire compared to the control SS. Understanding this variation will be clinically helpful during orthodontic treatment. Therefore, when giving long-duration CHX and NaF-based rinses to SS archwire patients, caution should be exercised. Sabah et al. also showed significant deterioration of SS orthodontic wires subjected to fluoride mouthwash, and the current study substantiates these facts. ^16^

This could be because fluoride-based mouthwash can disrupt the protective corrosion-resistant layer of chromium oxide on the surface of SS archwires, making them vulnerable to corrosion. ^5^ This leads to a phenomenon known as hydrogen embrittlement that causes increased hydrogen absorption in stainless steel, which can impact its mechanical properties when confined within the steel lattice. ^5, 10^ Further, according to Shibata et al., fluoride causes “stress corrosion cracking of SS archwire,” It was suggested that SS wire might interact with hydrofluoric acid and the potential degradation reaction (breaking of a passive layer) ^. 22^ Similarly, Omidkhoda et al. reported that the use of CHX resulted in intergranular corrosion on the surface of the bracket, which can cause discoloration, weakening of the archwire, and, finally, breakage. ^23^ Along similar lines, Aghili et al. substantiated the current results that represented that the application of CHX causes a significant reduction in MoE values of SS orthodontic archwire. ^2^

Conversely, the HiORA herbal mouthwash/organic extract-based solution used in the study did not cause any significant degradation in the mechanical properties of SS orthodontic archwires. There are negligible reports found in the literature on the effect of HiORA on orthodontic archwires. However, HiORA has been reported to be an excellent alternative mouthwash and was found to be more effective than a chlorine dioxide mouthwash in reducing total colony-forming units, gingivitis, and plaque accumulation. ^19, 24, 25^ In the present study, the values of MoE of SS archwire subjected to HiORA mouthwash are approximately equal to the control archwire sample. HiORA did not lead to any noteworthy alteration in the MoE values of SS orthodontic archwires.

### Nickel titanium orthodontic archwire

NiTi's shape memory and pseudoelasticity are advantageous in the initial alignment phase of orthodontic treatment. ^26^ In the present study, when the NiTi wires dipped into the NaF, there was a significant reduction in the modulus of elasticity during loading and unloading forces, and they needed slightly less loading force to deflect than the control. These results indicate that fluoride-based (NaF) mouthwashes adversely affected the mechanical property of NiTi orthodontic archwire. These results align with other reports that topical fluorides can cause corrosion in titanium-based orthodontic wires. ^3, 12– 15^ According to Walker et al. and other authors, Prevident and Phosflur gel (fluoride) significantly reduced the unloading MoE of NiTi wires. It has been observed that, like SS orthodontic archwire, fluoride exposure can result in the loss of the oxide layer of NiTi wires. This can lead to the exposure of the underlying alloy, resulting in its corrosion and subsequent assimilation of hydrogen ions due to the strong titanium-hydrogen attraction. ^4, 5, 10, 27, 28^ The hydrogen concentration in NiTi wire increases proportionally with immersion duration. Similarly, Watanabe and Watanabe found a significant decrease in NiTi mechanical properties along with a change in color and surface morphology after 1hr and 24 hrs immersion of NiTi into NaF solution. ^6^

Contrary to the effect of NaF on NiTi, when these wires were dipped into 0.2% Chlorhexidine mouthwash, they required slightly more force to deflect than the control wire, and MoE values increased during loading forces. However, it significantly decreased during unloading forces in the current study. However, Hosseinzadeh NT et al. found that immersing NiTi wire in 0.2% CHX mouthwash did not considerably change mechanical properties. ^29^ This aspect may be further investigated by applying CHX to NiTi archwire for a longer duration.

HiORA does not induce statistically significant changes in MoE values of NiTi orthodontic wire. There is no report on the effect of HiORA on NiTi archwires. Nonetheless, CHX and HiORA are safer mouthwashes for patient orthodontic treatment using NiTi archwire during the initial leveling and alignment phase.

### Beta titanium orthodontic archwire

β-Ti Orthodontic archwire has a lower MoE than SS and is nearly twice as strong as NiTi orthodontic alloys. Closing loops, gable bends, and attachments are employed in the intermediate stages of therapy. These requirements are met by beta-titanium wires, which have a more comprehensive activation range than SS wires. Furthermore, β -Ti has a similar corrosion resistance to SS. Walker et al. reported that exposure to preventive fluoride gels (acidulated or neutral) causes a noteworthy reduction in unloading mechanical properties. ^5^ Ogawa et al. stated that the increase of hydrogen absorption and mortification of mechanical property of β-Ti orthodontic wires increases with fluoride exposure. ^30, 31^ Similarly, this study also indicates the reduction of unloading MoE after exposure to CHX and NaF mouthwash. However, the herbal mouthwash HiORA has proven to be kind for long-term use in conjunction with beta titanium orthodontic archwire during a given clinical scenario. It was also noticed that although NaF and HiORA significantly needed more force to deflect the β-Ti archwires than the control, there was an insignificant change in loading MoE.

### Limitations

The present study design was In-vitro, where the orthodontic wires were subjected to mouthwashes for 1 minute/day for three months. However, the orthodontic archwire continuously interacts intraorally with saliva, beverages, food, medications, etc. The deterioration process started by the mouthwash might be further continued (at a similar or different rate). Attempts in the future may be made with the help of in vivo studies using conventional archwires of SS, NiTi, and β-Ti to corroborate present findings. In future studies, brackets of different materials and prescriptions may be combined with archwires. Attempts may be made to assess the time of prescription of a particular mouthwash associated with a specific stage of treatment.

## Conclusion

The present study aimed to discover the best mouthwash that can be prescribed routinely during orthodontic treatments using SS, NiTi, and β-Ti orthodontic archwires. The NaF-based solution/mouthwash is more corrosive in the case of SS and NiTi archwire and, therefore, should be prescribed carefully. It also caused a reduction of the unloading MoE values of beta titanium archwire and may be clinically relevant as unloading forces produce tooth movement; hence, it should be used cautiously. CHX also deteriorated the mechanical property of the SS orthodontic archwire and was found to harm NiTi and β-Ti archwires during unloading. Therefore, it should be cautiously prescribed in the finishing stage with NiTi and β-Ti. HiORA has proven to be the gentlest in all the categories analyzed in the present study. Thus, this herbal mouthwash can be safely prescribed during all stages of orthodontic treatment and clinical scenarios due to its negligible effect on the mechanical properties of conventional archwires during orthodontic treatment.

## Software availability statement

The software used in this study, [Statistical software SPSS version 29 and Blue Hill Software], are available for public access. It can be downloaded from https://www.ibm.com/account/reg/us-en/signup?formid=urx-19774 and https://instron-bluehill-universal.software.informer.com/download/.

## Ethical approval

The study was approved by the Kasturba Hospital Ethics Committee, Kasturba Hospital, Manipal, Karnataka (IEC 869/2019) on 13 ^th^ November 2019.

Consent not required since there was no human involvement in the study.

## Data Availability

Figshare: Evaluation of the effect of two conventional and one herbal mouthwash on the Modulus of Elasticity of three orthodontic wires: An in vitro study. Doi https://doi.org/10.6084/m9.figshare.27043408. ^32^ The project contains the following underlying data Sheet 1:Modulus of Elasticity-Loading samples. Sheet 2: Modulus of Elasticity-Unloading samples. Data are available under the terms of the Creative Commons Attribution 4.0 International license (CC-BY 4.0).
