# Supplementary material for: Evaluation of the effect of two conventional and one herbal mouthwash on the modulus of elasticity of three orthodontic wires: an in vitro study
Source: F1000Res. 2025 Nov 25;14:336. Originally published 2025 Mar 27. [Version 2] doi: 10.12688/f1000research.156706.2 (PMC12673257; doi:10.12688/f1000research.156706.2)
Supplement: Peer review history

# Reviewer report: Reviewer response for version 2

**DOI:** 10.5256/f1000research.191907.r435839

**Published:** 2025-12-02

**Authors:** Sarmad S. Salih Al Qassar

**Affiliations:** 1 University of Mosul, Mosul, Iraq

Dear editor,

 I have no further comments, the revised version is now suitable for indexing.

 Regards

Is the work clearly and accurately presented and does it cite the current literature?

Partly

If applicable, is the statistical analysis and its interpretation appropriate?

Yes

Are all the source data underlying the results available to ensure full reproducibility?

Yes

Is the study design appropriate and is the work technically sound?

Yes

Are the conclusions drawn adequately supported by the results?

Yes

Are sufficient details of methods and analysis provided to allow replication by others?

Yes

Reviewer Expertise:

Orthodontic materials, Curve of Spee. 3D analysis. Bite Force.

I confirm that I have read this submission and believe that I have an appropriate level of expertise to confirm that it is of an acceptable scientific standard.

# Reviewer report: Reviewer response for version 1

**DOI:** 10.5256/f1000research.172055.r427048

**Published:** 2025-11-10

**Authors:** Snehlata Oberoi

**Affiliations:** 1 University of California San Francisco, San Francisco, California, USA

Well designed study with important findings and conclusions that cautiously prescribed in the finishing stage with NiTi and β-Ti. Also,  herbal mouthwash can be safely prescribed during all stages of orthodontic treatment and clinical scenarios due to its negligible effect on the mechanical properties of conventional archwires during orthodontic treatment.

Is the work clearly and accurately presented and does it cite the current literature?

Yes

If applicable, is the statistical analysis and its interpretation appropriate?

Yes

Are all the source data underlying the results available to ensure full reproducibility?

Yes

Is the study design appropriate and is the work technically sound?

Yes

Are the conclusions drawn adequately supported by the results?

Yes

Are sufficient details of methods and analysis provided to allow replication by others?

Yes

Reviewer Expertise:

Orthodontics, Craniofacial

I confirm that I have read this submission and believe that I have an appropriate level of expertise to confirm that it is of an acceptable scientific standard.

# Reviewer report: Reviewer response for version 1

**DOI:** 10.5256/f1000research.172055.r374534

**Published:** 2025-04-16

**Authors:** Sarmad S. Salih Al Qassar

**Affiliations:** 1 University of Mosul, Mosul, Iraq

Dear Editor,

 Thank you for considering me as a reviewer for the manuscript titled *"Evaluation of the Effect of Two Conventional and One Herbal Mouthwash on the Modulus of Elasticity of Three Orthodontic Wires: An In Vitro Study."*

 The manuscript is well-written and well-structured, addressing an important topic related to the impact of different mouthwashes on the mechanical properties—specifically, the modulus of elasticity—of various orthodontic archwires used in fixed appliance treatment. However, I have several comments and suggestions for improvement:

**Comments:**

1. **Introduction:**
  - The introduction could be further developed to provide more depth. The authors should expand on the effects of the oral environment, including saliva and its pH, on fixed orthodontic appliances and vice versa.
  - I recommend incorporating discussions from the following article to enhance this section:
    Alkasso, I. R., et al (2021). [Ref-1].
  - In the third paragraph, more recent literature on the effects of different mouthwashes on the corrosion of orthodontic appliances should be cited. A relevant study to consider:
    Al Qassar, S. S. S., et al., (2024). [Ref-2].
2. **Methods Section:**
  - More clarification is needed regarding the sample size determination. How was the sample size calculated? Was a power analysis conducted?
  - The study did not include pH evaluation of the tested mouthwashes or thermocycling. While the authors acknowledged this as a limitation, they should provide a stronger justification for why these were omitted.
  - The incubation period of three months requires scientific justification. Why was this specific timeframe chosen? How does it correlate with clinical conditions?
3. **Results and Discussion:**
  - The authors should clarify the clinical implications of their findings. How do these results translate into real-world orthodontic practice?
  - The evaluation protocol for measuring the modulus of elasticity should be elaborated on to ensure reproducibility.
4. **Limitations:**
  - The limitations section should be expanded to cover additional drawbacks of the study design, such as the lack of thermocycling and pH assessment.

**References:**

- More updated references from high-quality, indexed journals ( *Scopus* and *Clarivate*) should be included.
- Avoid citing references from less reputable journals. For example, reference No. 14 should be reconsidered.

**Conclusion:**

 This study presents valuable insights into the effects of mouthwashes on orthodontic wires. However, addressing the above concerns will significantly improve the manuscript's quality and impact.

 Thank you for the opportunity to review this paper.

Is the work clearly and accurately presented and does it cite the current literature?

Partly

If applicable, is the statistical analysis and its interpretation appropriate?

Yes

Are all the source data underlying the results available to ensure full reproducibility?

Yes

Is the study design appropriate and is the work technically sound?

Yes

Are the conclusions drawn adequately supported by the results?

Yes

Are sufficient details of methods and analysis provided to allow replication by others?

Yes

Reviewer Expertise:

Orthodontic materials, Curve of Spee. 3D analysis. Bite Force.

I confirm that I have read this submission and believe that I have an appropriate level of expertise to confirm that it is of an acceptable scientific standard, however I have significant reservations, as outlined above.

## References

[1] : Evaluation of antiseptic mouthwashes protocol against SARS-CoV-2 on orthodontic appliances (an in vitro study). *Brazilian Dental Science* .2024;27(2) : 10.4322/bds.2024.e4196 10.4322/bds.2024.e4196

[2] : Durability of different types of Mouthwashes on the Salivary Buffering system in Orthodontic Patients. *Dentistry 3000* .2021;9(1) : 10.5195/d3000.2021.161 10.5195/d3000.2021.161
